# Comparative Genome Analysis of a Pathogenic *Erysipelothrix rhusiopathiae* Isolate WH13013 from Pig Reveals Potential Genes Involve in Bacterial Adaptions and Pathogenesis

**DOI:** 10.3390/vetsci7020074

**Published:** 2020-06-04

**Authors:** Longsheng Yang, Yongwei Zhu, Zhong Peng, Yi Ding, Kai Jie, Zijian Wang, Ying Peng, Xibiao Tang, Xiangru Wang, Huanchun Chen, Chen Tan

**Affiliations:** 1 State Key Laboratory of Agricultural Microbiology, College of Veterinary Medicine, Huazhong Agricultural University, Wuhan 430070, Hubei, China; 13813873804@139.com (L.Y.); hzau_work_zyw@163.com (Y.Z.); pengzhong@mail.hzau.edu.cn (Z.P.); dingyi@mail.hzau.edu.cn (Y.D.); jiekai@webmail.hzua.edu.cn (K.J.); wangzijian0313@163.com (Z.W.); pengying0613@163.com (Y.P.); wangxr228@mail.hzau.edu.cn (X.W.); chenhch@mail.hzau.edu.cn (H.C.); 2 Ministry of Education and Hubei Province Co-constructed Cooperative Innovation Center for Sustainable Pig Production, Huazhong Agricultural University, Wuhan 430070, Hubei, China; tangren77@126.com; 3 Chia Tai Animal Husbandry Investment (Beijing) Co., Ltd., Beijing 100005, China; 4 State Key Laboratory of Meat Processing and Quality Control, Jiangsu Yurun Meat Group Co., Ltd., Nanjing 210000, China; 5 Key Laboratory of Development of Veterinary Diagnostic Products of Ministry of Agriculture, Huazhong Agricultural University, Wuhan 430070, Hubei, China

**Keywords:** *Erysipelothrix rhusiopathiae*, PacBio RSII and Illumina sequencing, comparative genomics analysis, bacterial adaptions and pathogenesis, core and dispensable genes

## Abstract

*Erysipelothrix rhusiopathiae* is a common pathogen responsible for pig erysipelas. However, the molecular basis for the pathogenesis of *E. rhusiopathiae* remains to be elucidated. In this study, the complete genome sequence of the *E. rhusiopathiae* strain WH13013, a pathogenic isolate from a diseased pig, was generated using a combined strategy of PacBio RSII and Illumina sequencing technologies. The strategy finally generated a single circular chromosome of approximately 1.78 Mb in size for the complete genome of WH13013, with an average GC content of 36.49%. The genome of WH13013 encoded 1633 predicted proteins, 55 tRNAs, as well as 15 rRNAs. It contained four genomic islands and several resistance-associated genes were identified within these islands. Phylogenetic analysis revealed that WH13013 was close to many other sequenced *E. rhusiopathiae* virulent strains. The comprehensive comparative analysis of eight *E. rhusiopathiae* virulent strains, including WH13013, identified a total of 1184 core genes. A large proportion (approximately 75.31%) of these core genes participated in nutrition and energy uptake and metabolism as well as the other bioactivities that are necessary for bacterial survival and adaption. The core genes also contained those encoding proteins participating in the biosynthesis and/or the components of the proposed virulence factors of *E. rhusiopathiae*, including the capsule (*cpsA*, *cpsB*, *cpsC*), neuraminidase (*nanH*), hyaluronidase (*hylA*, *hylB*, *hylC*), and surface proteins (*spaA*, *rspA*, *rspB*). The obtaining of the complete genome sequence of this virulent strain, WH13013, and this comprehensive comparative genome analysis will help in further studies of the genetic basis of the pathogenesis of *E. rhusiopathiae*.

## 1. Introduction

*Erysipelothrix rhusiopathiae* is a non-spore forming, encapsulated, slightly rod-shaped, Gram-positive bacterium, and it is the causative agent of erysipelas, which manifests acute septicemia, diamond skin, and high fever [1]. Before 1990, erysipelas was one of the main fulminating infectious diseases in pigs in China [2,3]. It has caused the deaths of large numbers of pigs in the last 20 years, but because of normalized management, the disease has only occurred on small farms in China [3]. In recent years, the occurrence of erysipelas has increased, and outbreaks have been observed in sows and finishers in many large-scale farms, causing great economic losses [4]. In addition, the incorrect use and overuse of antimicrobials lead to the emergence and prevalence of field strains with increased drug resistance, which makes it more difficult to control the bacterium [3]. However, limited knowledge was gained on the pathogenesis of *E. rhusiopathiae*.

In recent years, with the generation of several *E. rhusiopathiae* genome sequences, some virulence factor encoding genes (VFGs) have been identified, including those responsible for the biosynthesis of capsule, surface proteins, antioxidant proteins, and phospholipase proteins [5]. The surface proteins mainly include protective antigens (SpaA, RspA and RspB) and hyaluronidase (HylA and HylB), which are related to biofilm formation and considered essential for resistance to phagocytosis and bacterial adherence [5,6,7,8]. The antioxidant proteins mainly consist of various reductases, which can help the organism escape from reactive oxygen species (ROS), and this is a major strategy for the intracellular survival of the organism [1]. The phospholipase proteins are composed of all kinds of phospholipases, which is beneficial to the organism to circumvent the host immune response.

The identification of virulence factors has become a hot spot in the field of studying *E. rhusiopathiae* [4]. However, the generation of multiple *E. rhusiopathiae* genome sequences has lagged behind many other bacterial pathogens. As of 31 October 2019, there are only 11 genome sequences of *E. rhusiopathiae* available on the National Center for Biotechnology Information (NCBI): these include eight complete sequences as well as three draft contigs (https://www.ncbi.nlm.nih.gov/genome/genomes/2104). The lack of multiple genome sequences of *E. rhusiopathiae* limited the identification of more genes associated with the bacterial pathogenesis and fitness. Previously, we characterized 48 *E. rhusiopathiae* clinical isolates from China using different methodologies, including pulse-field electrophoresis (PFGE) typing, antimicrobial susceptibility testing (AST), and virulence genotyping by PCR detection of the known VFGs [2]. In this study, we selected a high pathogenic *E. rhusiopathiae* strain, WH13013, from these 48 clinical isolates for the complete genome sequencing and comparative genomic analysis. The objective of this study was to identify the putative genes associated with the pathogenesis and fitness of *E. rhusiopathiae*.

## 2. Materials and Methods

### 2.1. Bacterial Strains

*E. rhusiopathiae* WH13013 was isolated from the spleen of a pig with a clinical history of acute septicemia in 2013 in Hubei, China [2]. The bacterium was maintained on Tryptic Toy Agar (Becton, Dickinson and Company, Franklin Lakes, NJ, USA) or Tryptic Soy Broth medium (Becton, Dickinson and Company, Franklin Lakes, NJ, USA) with 10% bovine serum (purchased from Hangzhou TIANHANG Company, Hangzhou, China) and grown to the mid-exponential phase at 37 °C under aerobic conditions.

### 2.2. Genome Sequencing, Assembly, and Annotation

Total genomic DNA was extracted using the Dneasy Blood & Tissue Kit (Qiagen, Dusseldorf, Germany). The concentration was determined by a Nanodrop2000 and the quality of the extracted total genomic DNA was evaluated by agarose gel electrophoresis. The complete genome sequence of WH13013 was determined by using the PacBio RS II plus the Illumina hybrid sequencing strategy at the Chinese National Human Genome Center in Shanghai, China. This combined strategy fully exploits the advantages of the long reads produced with third-generation sequencing and the high coverage of next-generation sequencing (NGS). Raw reads obtained with third-generation sequencing (about 0.9 Gb) and NGS (about 1.6 Gb) were combined for quality control. Approximately 1.1 Gb of high-quality data were finally obtained. The reads were then de novo assembled using the HGAP2 protocol on SMRT Portal v2.3.0 (https://www.pacb.com/documentation/smrt-analysis-software-installation-v2-3-0/). After assembly, a circular chromosome with a 630-folds average base coverage was finally generated. The final resulting genomes were annotated using the best-placed reference protein set (GeneMarkS+) in the NCBI Prokaryotic Genome Annotation Pipeline (version. 3.3) [9]. Predicted proteins were annotated using the NCBI non-redundant (NR) database, KEGG database [10], and/or COG database [11], with BLASTP (E-value < 1 × 10^−6^). The complete genome sequence as well as the annotated information were deposited in GenBank under the accession number CP017116.

### 2.3. Bioinformatical Analysis and Comparative Genomics

The genome plot of WH13013 was generated with the DNAPlotter tool [12]. The subcellular localization of the WH13013 proteins was predicted with PSORTb v.3.0.2 [13]. Insertion sequence (IS) elements, genome islands (GI), and prophage sequences were searched with IS Finder, IslandViewer 4 [14], and PHASTER [15], respectively. In order to identify virulence genes and drug resistance genes, the predicted amino acid sequences were compared with the virulence factor database (VFDB) [16] and the CARD database [17].

For comparative genomic analysis, the complete genome sequences of 8 *E. rhusiopathiae* strains, including Fujisawa (GenBank accession no. AP012027) [15], NCTC8163 (GenBank accession no. LR134439), ZJ (GenBank accession no. NZ_CP041995), GXBY-1 (GenBank accession no. NZ_CP014861), ML101 (GenBank accession no. NZ_CP029804), KC-Sb-R1 (GenBank accession no. NZ_CP033601), and SY1027 (GenBank accession no. CP005079) [16] were downloaded from GenBank (https://www.ncbi.nlm.nih.gov/genome/genomes/2104). Sequence comparisons were performed and visualized using BLAST Ring Image Generator (BRIG), with default parameters [18]. The average nucleotide identity between two genome sequences was calculated by using the ANI calculator [19]. Core genes were designated as those sharing 90% minimum DNA identity with 90% minimum alignment length [3]. Molecular phylogenetic analysis was performed by the maximum likelihood method based on the Tamura–Nei model [20] using MEGA X [21]. A phylogenetic tree was generated based on the nucleotide sequences of seven house-keeping genes (*gpsA*, *recA*, *purA*, *pta*, *prsA*, *galK*, *ldhA*) [22].

## 3. Result and Discussion

### 3.1. Overview of WH13013 Complete Genome Sequence

**Figure 1:**
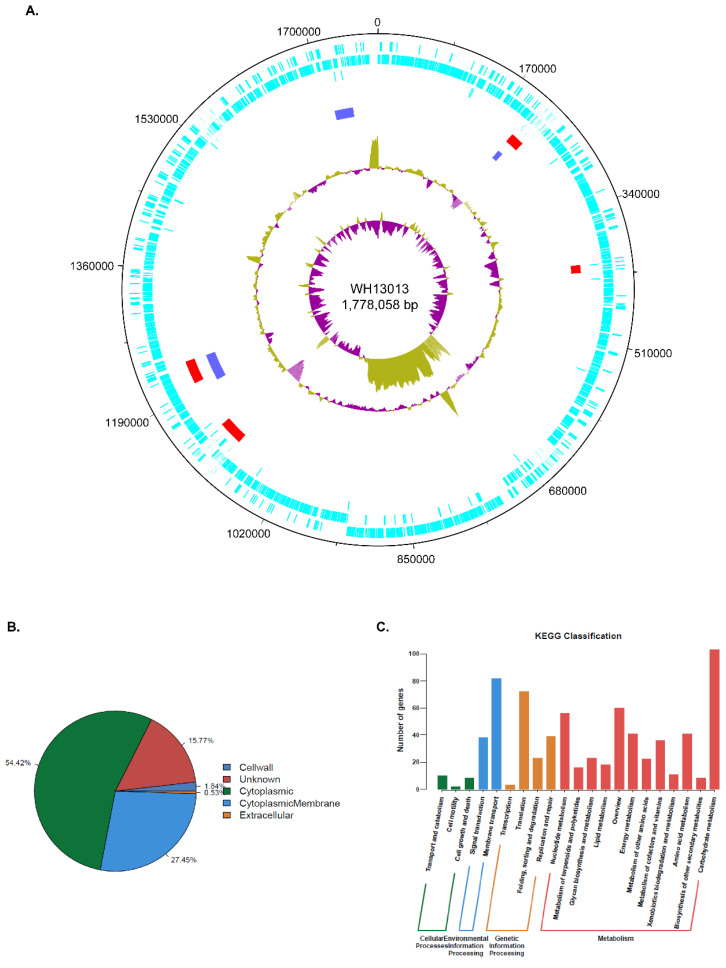
General features of the WH13013 complete genome. (**A**) Circular map of the complete genome of WH13013 which was generated via DNAPlotter. From the outside to the inside, the circle 1 represents the DNA base position (bp); circle 2 (cyan-blue), protein-coding regions transcribed clockwise; circle 3 (cyan-blue), protein-coding regions transcribed anti-clockwise; circle 4 (cyan-blue), predicted RNA genes; circle 5 (red), predicted genomic islands; circle 6 (blue), predicted phages sequences; the two innermost circles represent G + C content and GC skew, respectively. (**B**) Pie chart showing the proportions of the genes encoded by the WH13013 genome localizing in different places of the cells. (**C**) Bar graph showing the number of genes being assigned into different KEGG classifications.

The PacBio RS II plus the Illumina hybrid sequencing strategy generated a single circular chromosome of 1,778,058 bp in size for the WH13013 genome (Figure 1A). The GC content was determined as 36.49%, and a total of 1633 proteins, 55 tRNAs, as well as 15 rRNAs were predicted. There was little difference on the genome size, GC content, the numbers of coding proteins, and RNAs between the different *E. rhusiopathiae* genomes (Table 1). Among the 1633 proteins predicted, more than 50% of the predicted proteins localized in the cytoplasm (54.42%), followed by those localized in the cytoplasmic membrane (27.45%), the cell wall (1.84%), and the extracellular milieu (0.53%) (Figure 1B). Functionally, approximately 99.29%, 79.43%, and 52.60% of the predicted proteins were assigned into the NR database, COG database, and KEGG database, respectively (Figure 1C). The bioinformatical analysis revealed that WH13013 contained four genomic islands (GIs) (Figure 1A). Of particular note was the 33.6-kb GI (kb 1098.6~1132.3) (Figure 1A). This genomic island contained several genes encoding proteins that might have a role in mediating antimicrobial resistance, including a bacteriocin ABC transporter ATP-binding protein (ID: BC346_05500), a bacteriocin transporter (ID: BC346_05505), and a multidrug ABC transporter (ID: BC346_05565). In addition, many transposase coding genes were also found in this genomic island, suggesting that the acquisition of this island is associated with the activity of some mobile genetic elements (MGEs). It has been documented that genomic islands always harbor genes involved in virulence, antibiotic resistance, and/or other adaptations and generally play critical roles in bacterial pathogenicity [23,24]. Therefore, the presence of those genomic islands in WH13013 might be beneficial for its adaptions as well as the pathogenesis during infection. This could be also reflected by that all of the other sequenced *E. rhusiopathiae* virulent strains possess several numbers of genomic islands (Table 1). In addition to genomic islands, the prophages in bacterial genomes might also have an effect on the virulence of hosts as well as contribute to the mobilization of the DNA content and influence the short-term evolution of pathogenic bacteria [25,26]. The prediction of prophages revealed that WH13013 contained three possible prophages (Figure 1A). Interestingly, two of the prophages were located within two of the genomic islands predicted (Figure 1A).

### 3.2. Phylogenetic Analysis of WH1303

**Figure 2:**
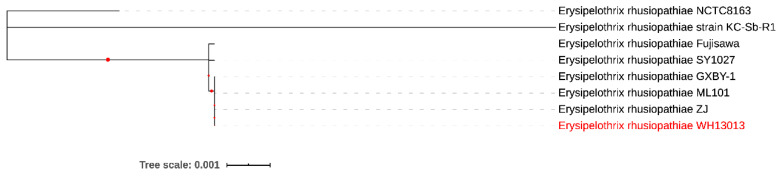
Phylogenetic analysis of different *Erysipelothrix rhusiopathiae*. The evolutionary history was inferred by using the maximum likelihood method and Tamura–Nei model. The tree with the highest log likelihood (−10,888.34) is shown. The percentage of trees in which the associated taxa clustered together is shown next to the branches. Initial tree(s) for the heuristic search were obtained automatically by applying neighbor-join and BioNJ algorithms to a matrix of pairwise distances estimated using the maximum composite likelihood (MCL) approach, and then selecting the topology with a superior log likelihood value. The tree is drawn to scale, with branch lengths measured in the number of substitutions per site. This analysis involved 8 nucleotide sequences. There was a total of 7323 positions in the final dataset. Evolutionary analyses were conducted in MEGA X.

Currently, there are only 11 genome sequences of *E. rhusiopathiae* available on the NCBI and 8 of them are complete sequences. Phylogenetic analysis revealed that WH1303 was closest to *E. rhusiopathiae* strains ZJ (GenBank accession no. NZ_CP041995), ML101 (GenBank accession no. NZ_CP029804), and GXBY-1 (GenBank accession no. NZ_CP014861) [27] (Figure 2). These four isolates from China were also close to *E. rhusiopathiae* strains Fujisawa (GenBank accession no. AP012027), which was isolated from Japan [28], and SY1027 (GenBank accession no. CP005079), which was also isolated from China [3] (Figure 2). These six isolates were far from *E. rhusiopathiae* strains NCTC8163 (GenBank accession no. LR134439), which was isolated from the United Kingdom, and KC-Sb-R1 (GenBank accession no. NZ_CP033601), which was isolated from South Korea.

### 3.3. Comparative Genome Sequence Analysis of the WH13013 Genome

**Figure 3:**
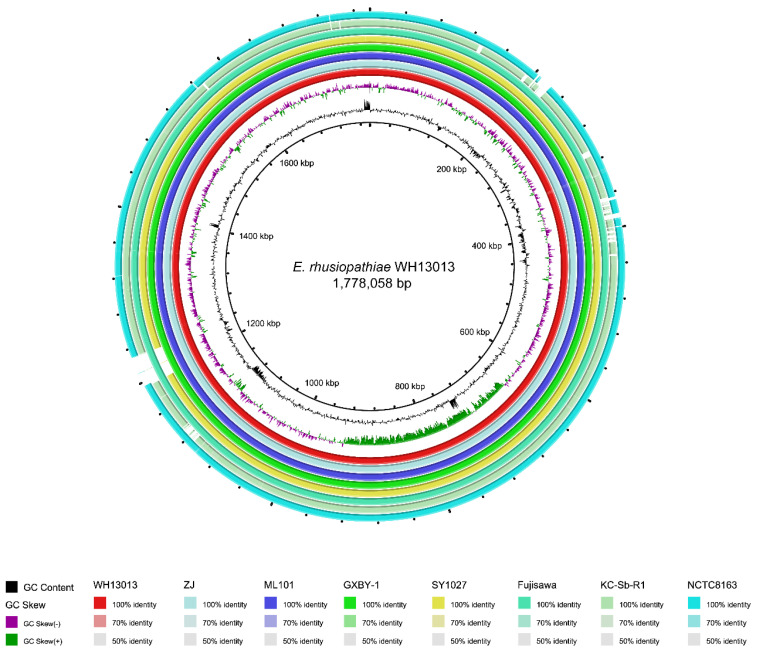
Comparisons of genomes related to WH13013. Shown is a BRIG circular plot for BLASTN comparisons of WH13013 and related genomes. The first and second inner rings represent the GC content and GC skew of WH13013, respectively. From the third ring inside, each ring corresponds to a different genome, as follows, from inner to outer ring: WH13013, ZJ, ML101, GXBY-1, SY1027, Fujisawa, KC-Sb-R1, and NCTC8163.

Comparative genomics analysis revealed that the genome sequence of WH13013 was highly homologous to that of ZJ (average nucleotide identity 99.99%), ML101 (average nucleotide identity 99.99%), GXBY-1 (average nucleotide identity 100.00%), Fujisawa (average nucleotide identity 99.98%), SY1027 (average nucleotide identity 99.94%), KC-Sb-R1 (average nucleotide identity 96.24%), and NCTC8163 (average nucleotide identity 99.05%) (Figure 3). However, the genomes of SY1027, KC-Sb-R1, and NCTC8163 lacked several areas that were harbored by the genome of WH13013 (Figure 3). In particular, a large area in WH13013 (approximately 36.5 kb, from kb 1193.2~1229.7) was missed in the genomes of SY1027, KC-Sb-R1, and NCTC8163 (Figure 3). This 36.5 kb region was adjacent to a tRNA (tRNA^Trp^) and it contained 44 predicted genes encoding phage packaging and structural proteins, lysin-holin, as well as hypothetical proteins. The bioinformatical analysis revealed that this region was a putative phage which was homologous to phage SE-1 (GenBank accession no NC_029078), a temperate bacteriophage infecting *E. rhusiopathiae* [29]. A previous study has found that a recombinant CHAP domain-containing protein encoded by SE-1 can effectively lyse host bacterial strains [29], and this protein (ID: BC346_05905) was also found in the phage area of WH13013, where it might be used as a good antibacterial agent.

In addition to this 36.5-kb phage area, WH13013 also had a 37.1-kb region (kb 1193.4~1230.5) which is missing in KC-Sb-R1 (Figure 3). This region was also adjacent to a tRNA (tRNA^Leu^) and it contained 18 predicted genes encoding an IS3 family transposase, an integrase, ATP transporters, and hypothetical proteins, suggesting the acquisition of this region might be associated with the activity of mobile genetic elements (MGEs). Of particular note is the presence of the radical S-adenosyl-l-methionine (SAM) protein (ID: BC346_05545) in this area. The radical SAM protein is a group of iron-sulfur containing proteins that exploit the reactivity of the high energy intermediate, the 5’-deoxyadenosyl radical, which is produced by reductive cleavage of SAM, to carry-out complex radical-mediated transformations [30]. In microorganisms, the activity of the radical SAM protein is very important for bacterial adaption to environmental niches and survival [31,32]. Another area carried by WH13013 but missing in KC-Sb-R1 was a 19.5-kb area (kb 203.8~223.3), which was also absent in NTCT8163 (Figure 3). This area contained 21 genes encoding an IS3 family transposase ISErh6, a restriction enzyme, a N-6 DNA methylase, a cell division protein FtsK, a DUF3173 domain-containing protein, a site-specific integrase, and several hypothetical proteins. The presence of genes encoding transposase, integrase, and methylase suggests the acquisition of this region might be also associated with the activity of MGEs. Another noteworthy area presented in WH13013 but missing in both KC-Sb-R1 and NTCT8163 was a 5.3-kb region (kb 384.2~389.5) (Figure 3). This region contained six genes encoding a glycerol-3-phosphate cytidylyltransferase (ID: BC346_01980), a CDP-Glycerol/Poly (glycerophosphate) glycerophosphotransferase (ID: BC346_01985), and two glycosyl transferases (ID: BC346_01985 and BC346_02005). This area is involved in the synthesis of teichoic acid, which has been reported to be beneficial for bacteria growing in phosphate-replete conditions [33,34].

### 3.4. Determination of Core and Dispensable Genes

**Figure 4:**
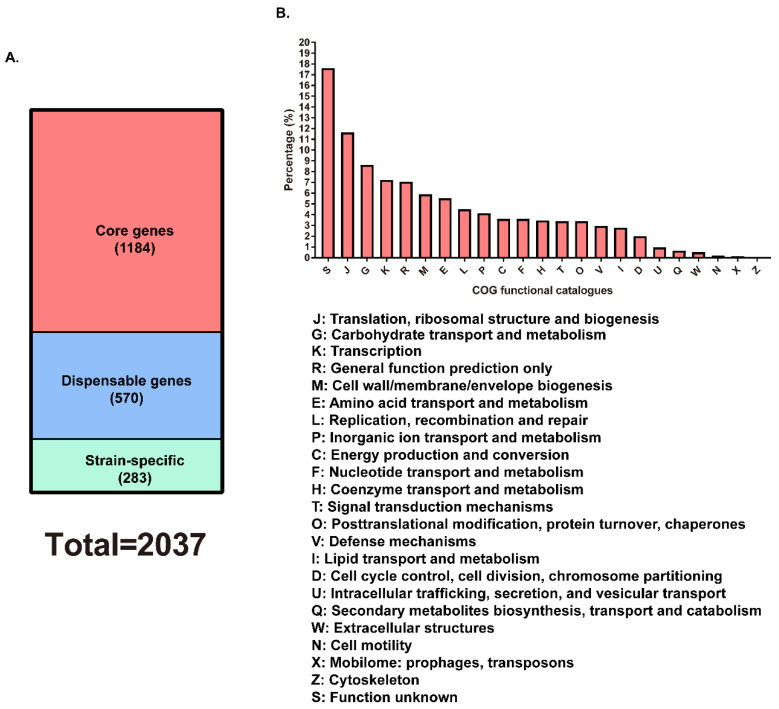
Distribution of core and dispensable genes among the eight *E. rhusiopathiae* virulent strains. (**A**) Distribution of core and dispensable genes. (**B**) COG functions of the core genes predicted.

As the eight *E. rhusiopathiae* strains with complete genome sequences publicly available in GenBank (Table 1), including WH13013, are virulent strains, analyzing the core genes as well as their functions will facilitate a good way to understand the genetic basis for the pathogenesis of WH13013 and the other *E. rhusiopathiae* strains. Therefore, the core genes shared by the eight *E. rhusiopathiae* virulent strains listed in Table 1 were determined using BLASTN with 90% DNA identity plus 90% alignment length as the minimum criterion. A total of 13,048 genes encoded by the eight *E. rhusiopathiae* strains were clustered and 1184 core genes, 570 dispensable genes as well as 283 strain-specific genes were identified (Figure 4). According to the COG functional prediction, a large proportion (approximately 75.31%) of core genes participated in the translation, ribosomal structure and biogenesis (J), carbohydrate transport and metabolism (G), transcription (K), cell wall/membrane/envelope biogenesis (M), amino acid transport and metabolism (E), replication, recombination and repair (L), inorganic ion transport and metabolism (P), energy production and conversion (C), nucleotide transport and metabolism (F), coenzyme transport and metabolism (H), signal transduction mechanisms (T), posttranslational modification, protein turnover, chaperones (O), defense mechanisms (V), and the other bioactivities that are necessary for bacterial survival and adaptions (Figure 4). The core genes also contained those encoding proteins participating in the biosynthesis and/or the components of the proposed virulence factors of *E. rhusiopathiae*, including the capsule (*cpsA*, *cpsB*, *cpsC*), neuraminidase (*nanH*), hyaluronidase (*hylA*, *hylB*, *hylC*), and surface proteins (*spaA*, *rspA*, *rspB*) [1,5,6]. The presence of these genes was undoubtedly beneficial for the fitness and pathogenesis of *E. rhusiopathiae* during the infection.

### 3.5. Determination of Antibiotic Resistance Genes

**Figure 5:**
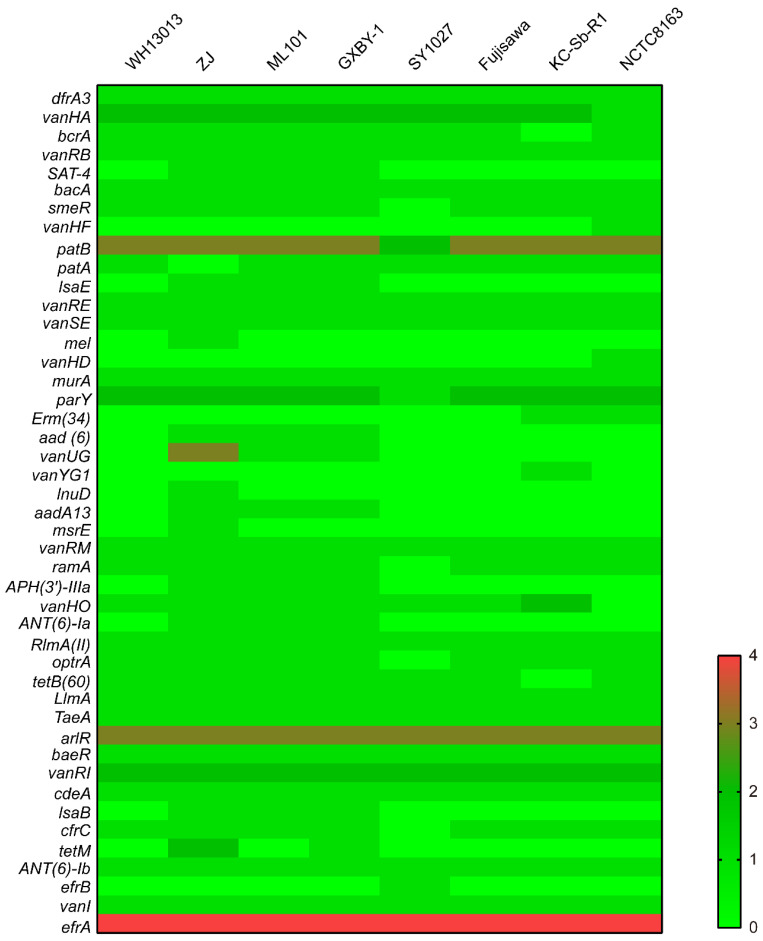
Heatmap showing the distribution of predicted antibiotic resistance genes in different *E. rhusiopathiae* strains. Copies of resistance genes found in each of the genomes were shown in different shades of colors.

The prediction of antibiotic resistance genes using the complete genome sequences revealed that WH13013 contained genes conferring putative resistance to multiple drugs including trimethoprim (*dfrA3*), peptide antibiotics (*bcrA*, *bacA*), glycopeptide antibiotics (*vanHA*, *vanRB*, *vanRE*, *vanSE*, *vanRM*, *vanHO*, *vanRI*, *vanI*), fluoroquinolones (*patB*, *patA*, *ramA*, *arlR*, *cdeA*, *efrA*), β-lactams (*smeR*, *ramA*, *efrA*), cephamycins (*smeR*, *ramA*), aminoglycosides (*smeR*, *baeR*, *ANT(6)-Ib*), penams (*smeR*, *ramA*), fosfomycins (*murA*), aminocoumarins (*parY*), tetracyclines (*ramA*, *optrA*, *tetB(60)*), macrolide antibiotics (*RlmA(II)*, *optrA*, *cfrC*, *efrA*), and phenicol antibiotics (*optrA*, *cfrC*) (Figure 5). The presence of these genes supports the phenotypes that WH13013 was resistant to tetracycline, cefazolin, lincomycin, gentamicin, kanamycin, sulfadiazine, and amikacin [2]. In addition, most of these resistance genes were also the broad characteristics of the other *E. rhusiopathiae* strains investigated in this study (Figure 5). Their presence may lead to treatment failure and contribute to the survival as well as the pathogenesis of *E. rhusiopathiae*.

### 3.6. Determination of Virulence Factors Coding Genes

**Figure 6:**
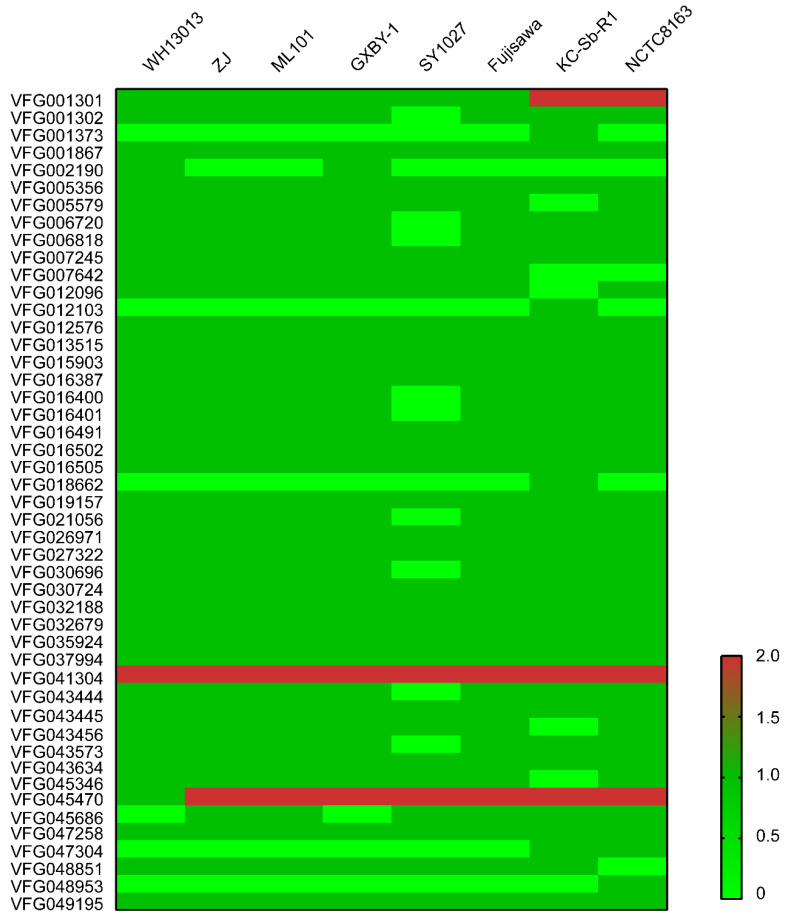
Heatmap showing the distribution of predicted virulence factor-associated genes in different E. rhusiopathiae strains. Copies of resistance genes found in each of the genomes were shown in different shades of colors.

Our previous study has determined that WH13013 contained many known virulence factors coding genes (VFGs), including *cpsA*, *cpsB*, *cpsC*, *spaA*, *rspA*, *rspB*, *hylA*, *hylB*, *hylC*, *nanH*, *pldB*, a patatin-like phospholipase A coding gene (ERH-0072), a phospholipase D coding gene (ERH-0388), a putative adhesin coding gene (ERH-1356), a cardiolipin synthetase coding gene (ERH-0333), a patatin-like phospholipase B coding gene (ERH-0334), and a lysophospholipase C coding gene (ERH-1433) [2]. The products of these VFGs are involved in the biosynthesis of capsule (*cpsA*, *cpsB*, *cpsC*), neuraminidase (*nanH*), hyaluronidase (*hylA*, *hylB*, *hylC*), surface proteins (*spaA*), fimbriae, and other adhesions (*rspA*, *rspB*) [1,5]. The prediction of VFGs revealed that WH13013 also contained many another putative VFGs that contributed to the fitness and pathogenesis, including those others that participated in the biosynthesis of capsule (VFG001301, VFG001302, VFG002190, VFG048851, VFG007642, VFG016400), fimbriae, and other adhesions (VFG043444, VFG043445, VFG043634, VFG016502, VFG016505), as well as the other possible virulence factors such as the elongation factor Tu (VFG016491), iron uptake, and iron acquisition proteins (VFG007245) (Figure 6, Table S1 in Supplementary Materials). Most of these VFGs were also presented in other *E. rhusiopathiae* virulent strains (Figure 6), suggesting that these VFGs are important for the bacterial adaption and pathogenesis. Indeed, capsule plays a key role in the resistance to phagocytosis and intracellular survival of *E. rhusiopathiae* [35,36,37]; surface proteins including fimbriae and other adhesions are as important in bacterial adherence to host cells during the infection [5]; iron is an essential element for bacterial survival and acts as an environmental signal that regulates the expression of many virulence factors [26,38].

## 4. Conclusions

In summary, we have sequenced the complete genome of *E. rhusiopathiae* WH13013, a pathogenic isolate from the spleen of a diseased pig in China. By comparison of the complete genomes of the other sequenced *E. rhusiopathiae* virulent strains, we analyzed the genomic biology of this bacterium and identified a series of genes that might contribute to its adaptions and pathogenesis during the infection. For example, the presence of multiple antibiotic resistance genes and the virulence factors encoding genes might increase the bacterial antibiotic resistance and virulence during the infection. Since the generation of multiple *E. rhusiopathiae* genome sequences has lagged behind many other bacterial pathogens, the obtaining of the complete genome sequence of this virulent strain, WH13013, and this comprehensive comparative genome analysis will help in further studies of the genetic basis of the pathogenesis of *E. rhusiopathiae*.

## Figures and Tables

**Table 1:** General features of the WH13013 genome and the other *E. rhusiopathiae* genomes.

Strain	GenBank Accession	Place of Isolation	Genome Size (bp)	G + C Content (%)	Proteins	tRNAs	rRNAs	No.s of Genomic Island	Prophage
WH13013	CP017116	China/Hubei	1,778,058	36.5	1633	55	15	4	3
ZJ	NZ_CP041995	China/Sichuan	1,945,689	36.5	1785	55	18	13	2
ML101	NZ_CP029804	China/Hunan	1,854,248	36.4	1699	55	15	10	1
GXBY-1	NZ_CP014861	China/Guangxi	1,876,490	36.5	1712	57	27	8	1
SY1027	CP005079	China/Jiangsu	1,752,910	36.4	1388	53	10	4	1
Fujisawa	AP012027	Japan	1,787,941	36.6	1633	55	21	5	3
KC-Sb-R1	NZ_CP033601	Korea/South sea	1,771,674	36.6	1599	55	21	3	2
NCTC8163	LR134439	United Kingdom/London	1,770,411	36.6	1609	55	27	5	2

## Data Availability

The complete genome of *E. rhusiopathiae* strain WH13013 was submitted to NCBI Genbank and is publicly accessible [GenBank:CP017116.1, BioProject: PRJNA331066, BioSample: SAMN05437124].

## Supplementary Materials

The following are available online at https://www.mdpi.com/2306-7381/7/2/74/s1; Table S1 Predicted functions of the virulence factors coding genes (VFGs).
